# Supplementary material for: Exploring neural entrainment and synchrony in response to repeated 60 Hz flickering white light in healthy volunteers
Source: PLoS One. 2025 Oct 7;20(10):e0332310. doi: 10.1371/journal.pone.0332310 (PMC12503310; doi:10.1371/journal.pone.0332310)
Supplement: S1 Table — (DOCX) [file pone.0332310.s001.docx]

| **Inclusion Criteria** | - Participant is male or female or diverse, between the ages of 18 – 65 - Participant is willing and able to sign informed consent form - Participant has signed informed consent form |
| --- | --- |
| **Exclusion Criteria** | - Participants who are pregnant. - Diagnosis of neurodegenerative diseases or psychiatric or brain injury/stroke, in particular:   - - -history of seizure or epilepsy     - -known diagnosis of migraine     - -tinnitus - Active treatment with Clopidrogel - Photosensitivity - Retinal diseases or cataract - Autoimmune diseases - Any skin lesion or skull morphology that might interfere with proper EEG reading - Participants with a work relationship with the Siegert lab or any of the sponsor cofounders |

**Supplementary Table 1 (S1)**

**Inclusion and exclusion criteria**
