# Supplementary figures and images for: Exploring neural entrainment and synchrony in response to repeated 60 Hz flickering white light in healthy volunteers
Source: PLoS One. 2025 Oct 7;20(10):e0332310. doi: 10.1371/journal.pone.0332310 (PMC12503310; doi:10.1371/journal.pone.0332310)

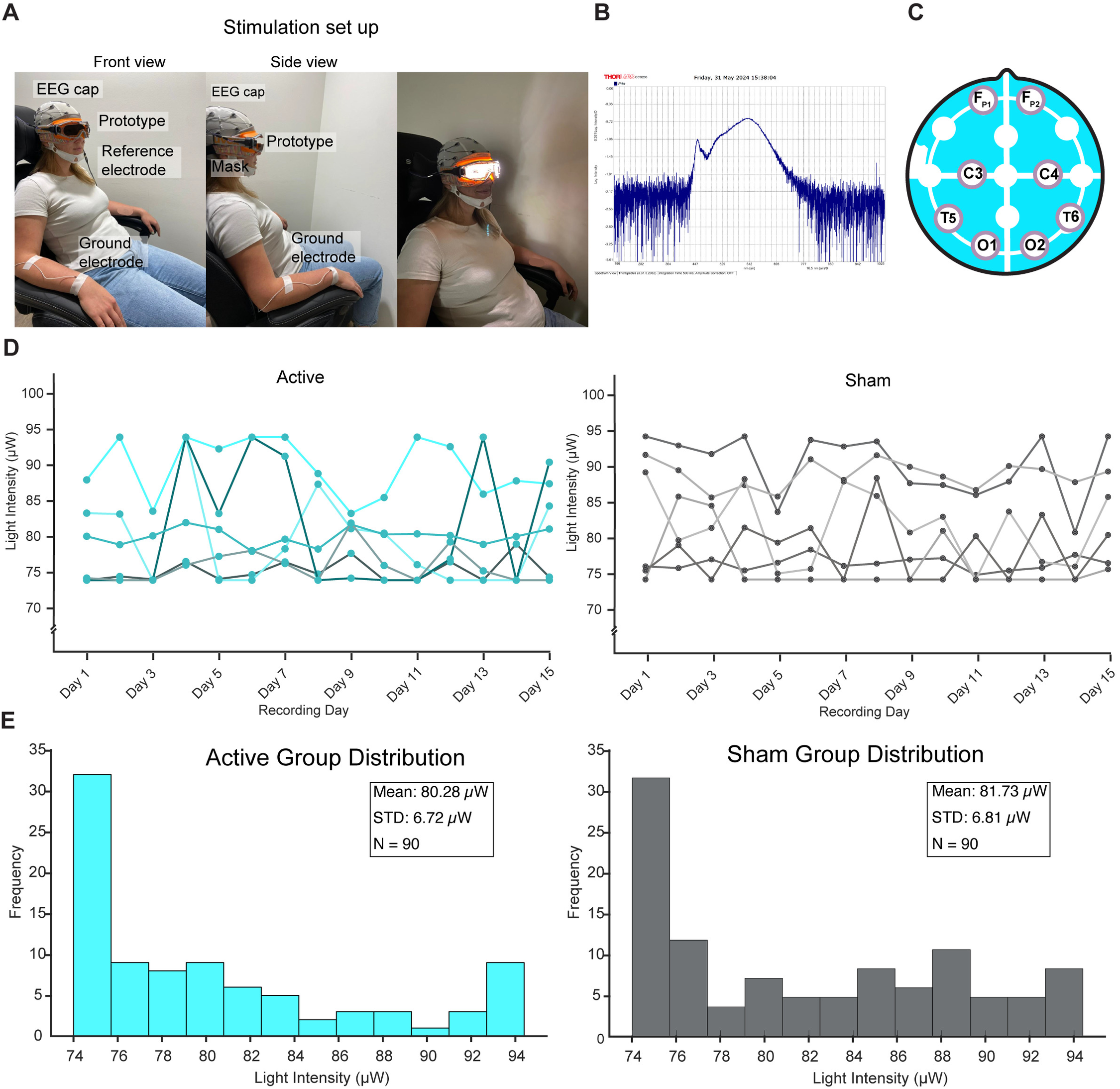

Supplement: S1 Fig — (A) Photographs of the experimental setup on EEG days: subjects were seated on a chair during the stimulation (picture was taken of one of the authors as an example). (B) Graph showing the light spectrum of the LEDs ranging from 440 nm to 770 nm, similar to daylight wavelengths. (C) The figure illustrates the location of the 8 EEG electrodes utilized in the study, mapped according to the 10–20 electrode placement system. (D) Analysis of individual light intensities chosen by participants (mean ± SD) across all sessions (15 days – 3 weeks, 5 days a week) and their distribution, demonstrating stable intensity settings within the comfort range (74–94 μW). (TIF) [file pone.0332310.s004.tif]

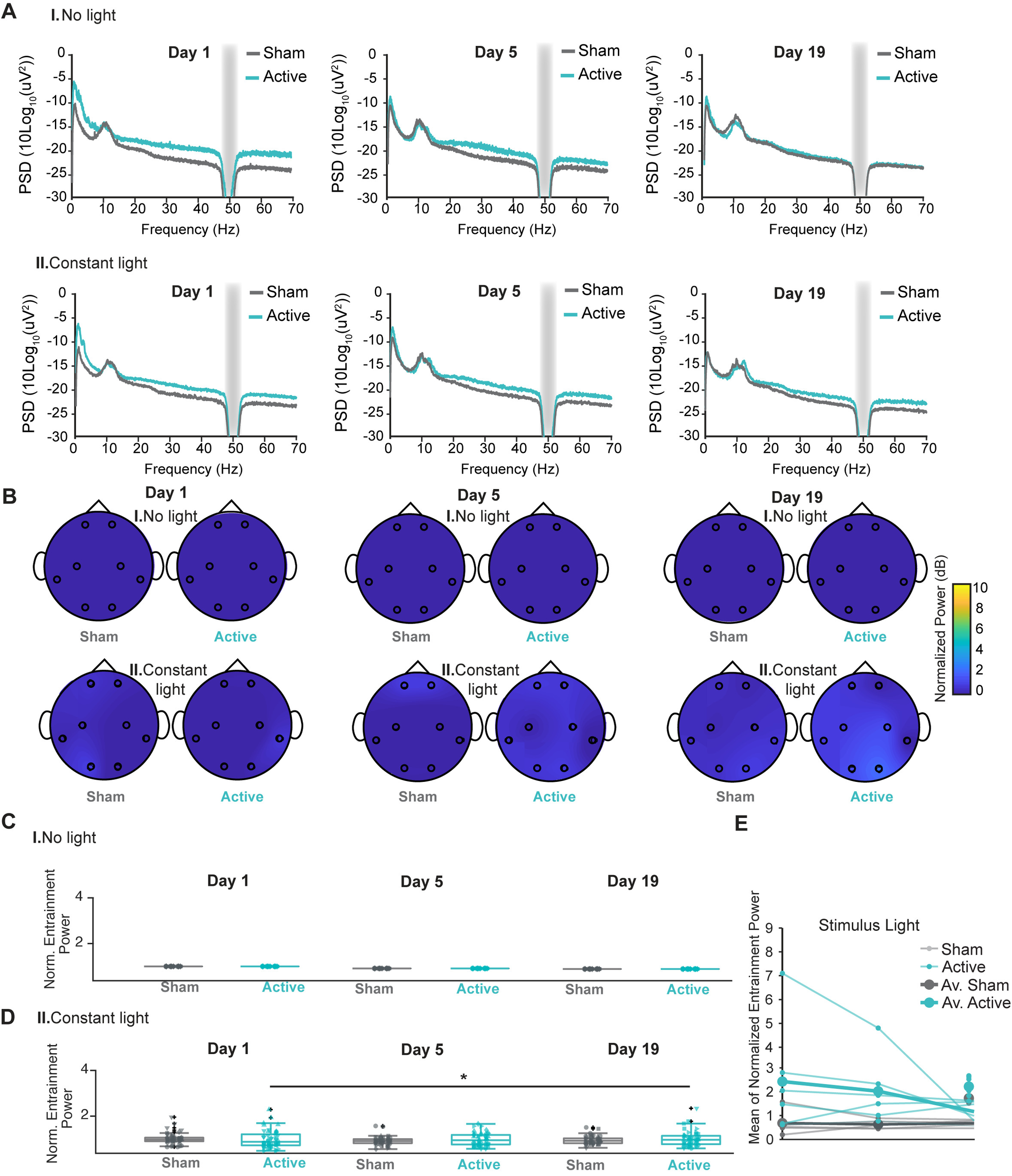

Supplement: S2 Fig — No light and II. Constant light stimulation (A) Scalp EEG power spectral density (PSD) averaged across all channels for participants in each group under (I.) No light and (II.) Constant light conditions. The gray bar indicates the 50 Hz line noise, which was notch-filtered. (B) Topographic maps showing normalized changes in 60 Hz PSD (relative to No light) averaged across participants of each group under (I.) No light and (II.) Constant light conditions. (C, D) 60 Hz PSD values across all channels between the active and sham groups under (I.) No light and (II.) Constant light conditions on days 1, 5, and 19. The observed statistically significant difference between Constant light condition of Active group day 1 and Active group day 19 is driven primarily by the large sample size (n), rather than any meaningful biological variation, as the average normalized 60 Hz power across these two days remains comparable. Statistical significance for these inter-group comparisons was assessed using the Wilcoxon rank-sum test. Furthermore, within the active group, significant differences in normalized PSD were detected between day 1 vs. day 19, accounting for repeated measurements. These intra-group comparisons across days were evaluated using the Kruskal-Wallis test, followed by post-hoc pairwise comparisons performed using Dunn’s test with Bonferroni correction. The normality of the data was assessed using the Shapiro-Wilk test, and non-parametric methods were applied due to deviations from normality. In this figure, different subjects are shape-coded. Only significant differences are indicated, with * indicating p < 0.05. Detailed p-values are provided in S3 Table. (E) The figure displays the average normalized PSD of all channels for each subject in both groups over days 1, 5, and 19, represented as individual lines. The thick line indicates the group average across all channels of all subjects. Data were recorded during light stimulation, with 60 Hz flickering light for the [file pone.0332310.s005.tif]

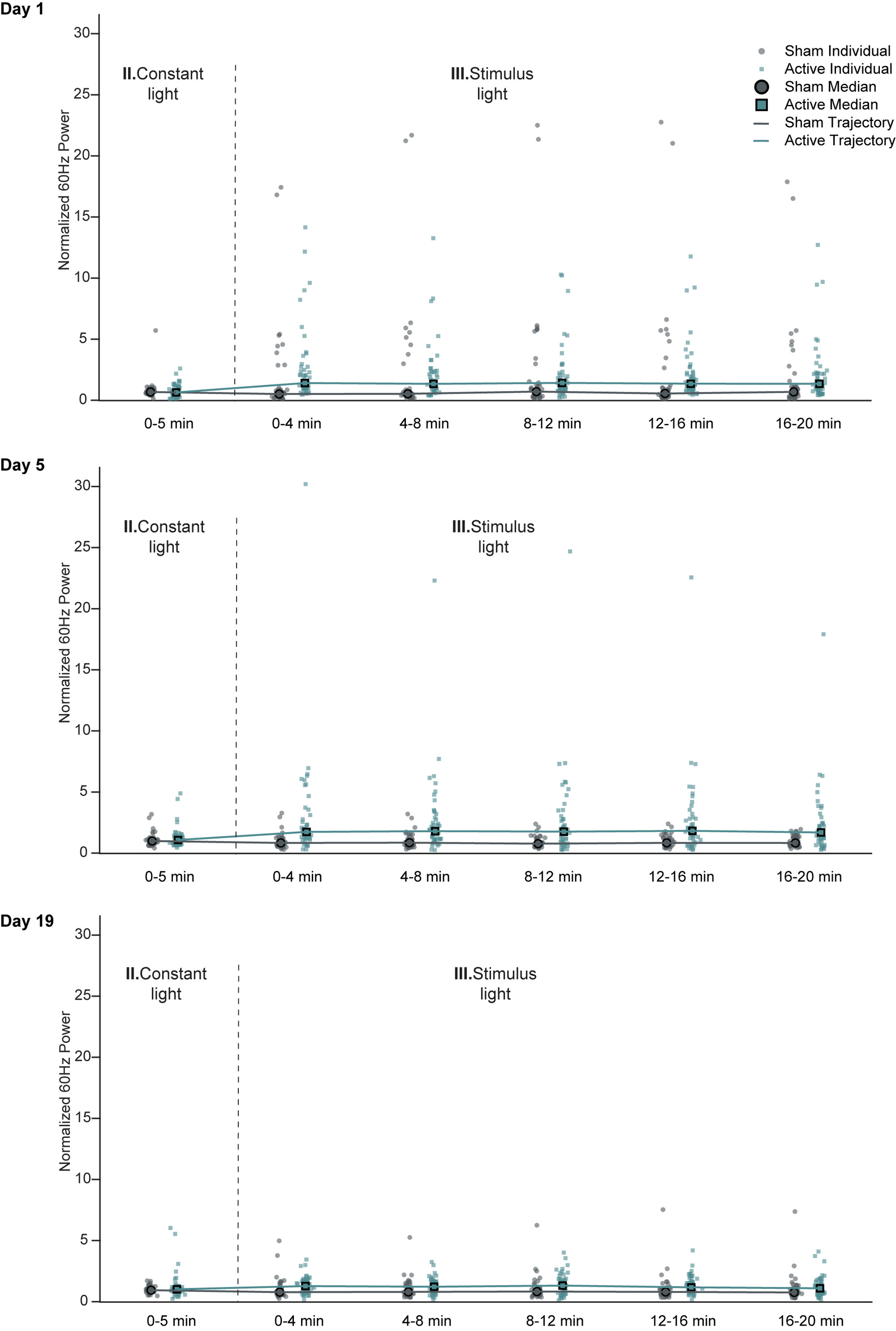

Supplement: S3 Fig — Each dot represents the normalized power spectral density (PSD) at 60 Hz from an individual EEG channel of a subject, normalized relative to the “No light” baseline. The line shows the trajectory of the group median across time points during each session. The stimulation phase, corresponding to the “Stimulus Light” condition, spans 20 minutes and is divided into four-minute time bins (0–4, 4–8, 8–12, 12–16, and 16–20 minutes). Normalized 60 Hz PSD values from all channels are averaged for each subject and then grouped by experimental condition (active vs. sham) on Days 1, 5, and 19. The figure illustrates how 60 Hz entrainment evolves temporally across sessions, highlighting the stability of 60 Hz entrainment over a 20 minutes stimulation period. (TIF) [file pone.0332310.s006.tif]

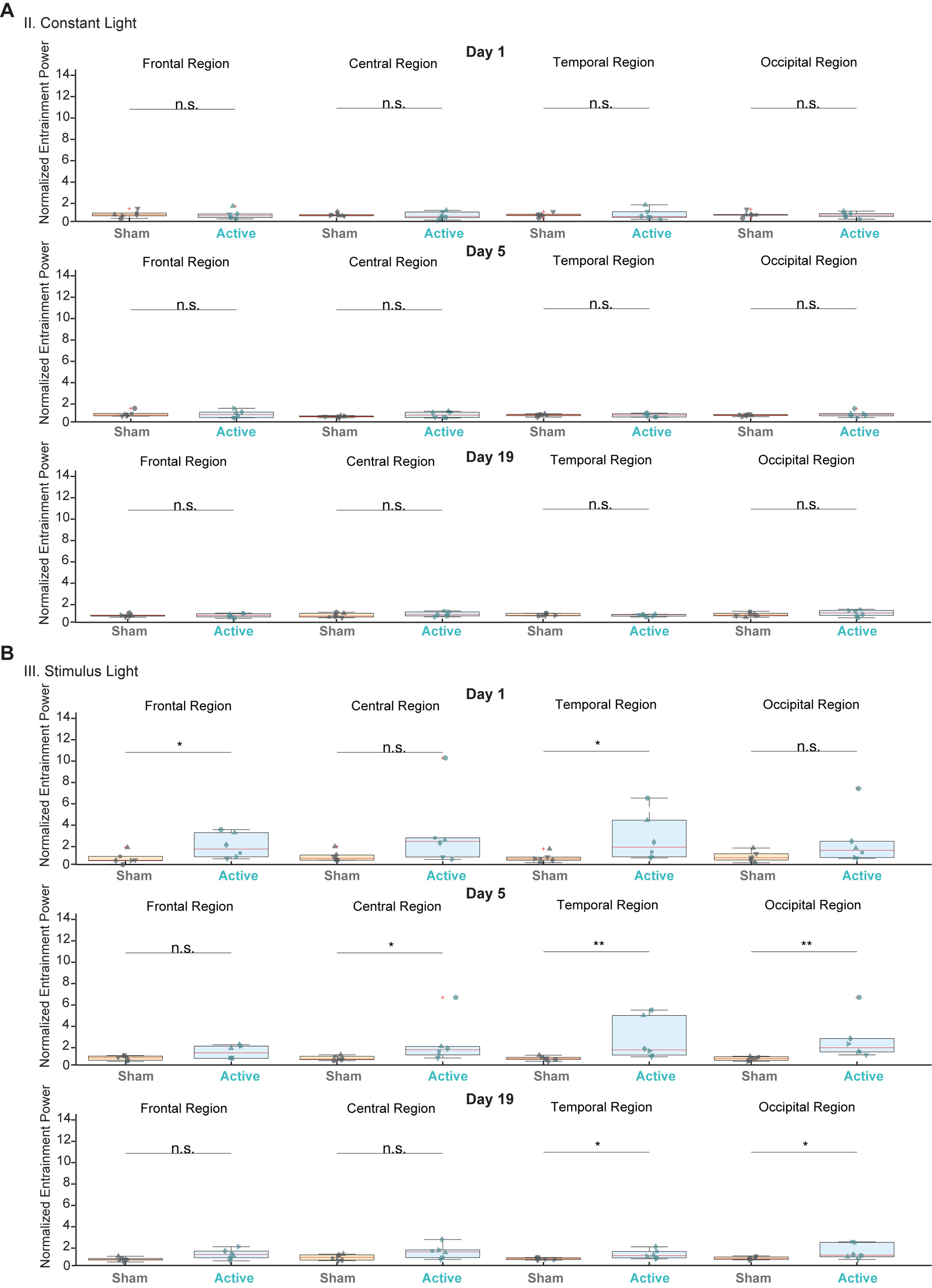

Supplement: S4 Fig — (A) Box plots depicting normalized 60 Hz power spectral density (PSD) relative to no light condition, averaged over electrode clusters representing the frontal (Fp1, Fp2), temporal (T5, T6), central (C3, C4), and occipital (O1, O2) regions, in the active and sham groups, across days 1, 5, and 19. Significant differences are indicated with *p < 0.05, **p < 0.01, and ***p < 0.001. Detailed p-values are reported in S3 Table. (TIF) [file pone.0332310.s007.tif]

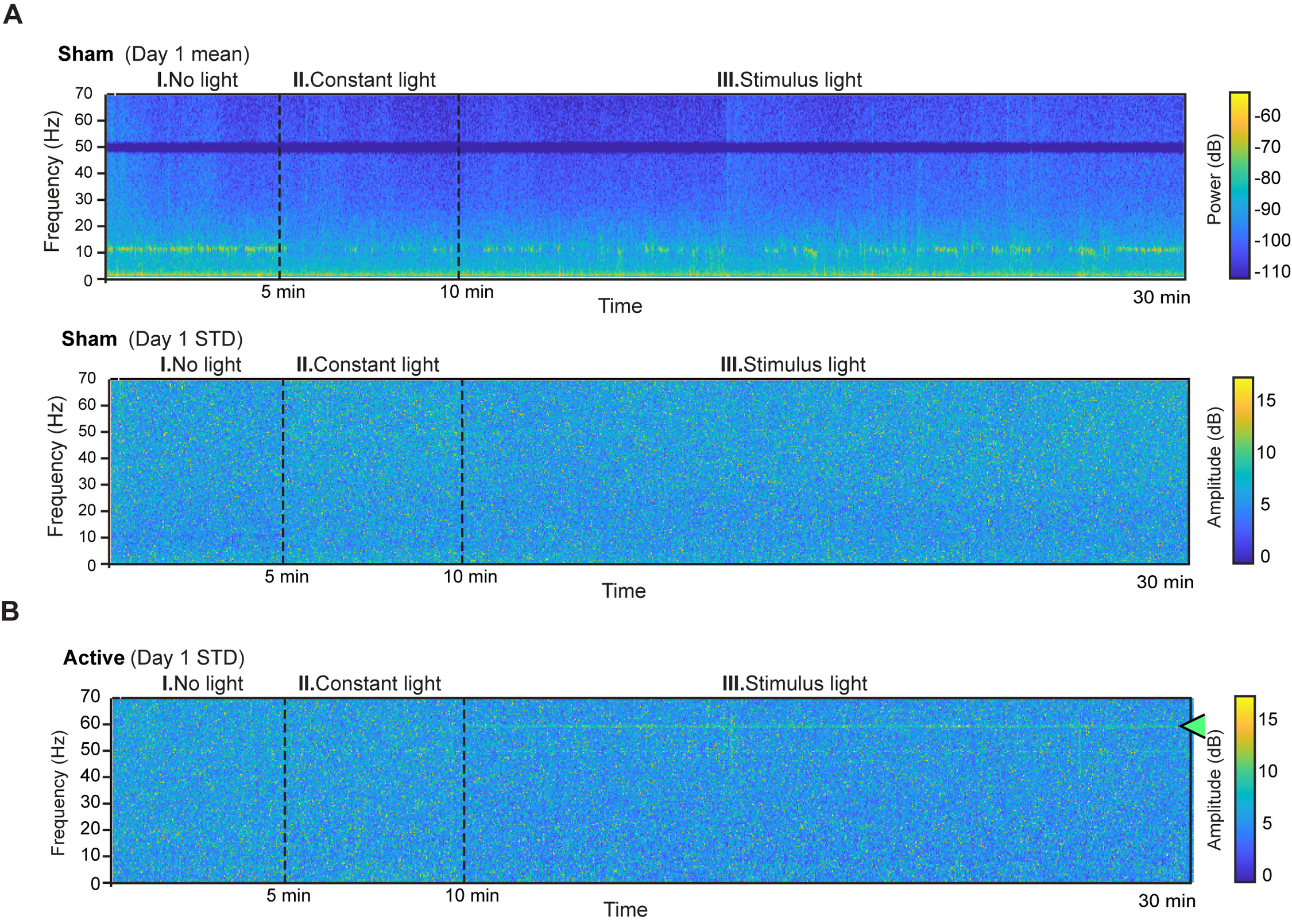

Supplement: S5 Fig — (A) Average STFT time-frequency plots for a representative sham participant (averaged across all electrodes) illustrate the absence of a visible 60 Hz signal during constant light exposure. (B) The standard deviation (SD) of the STFT power at 60 Hz for the same representative sham participant confirms low variability and no entrainment at the stimulation frequency. (C) Standard deviation (SD) of STFT power at 60 Hz across all electrodes for a representative active group participant on day 1. The green arrow indicates variability in the 60 Hz entrainment due to the variability in the level of entrainment in different channels. (TIF) [file pone.0332310.s008.tif]

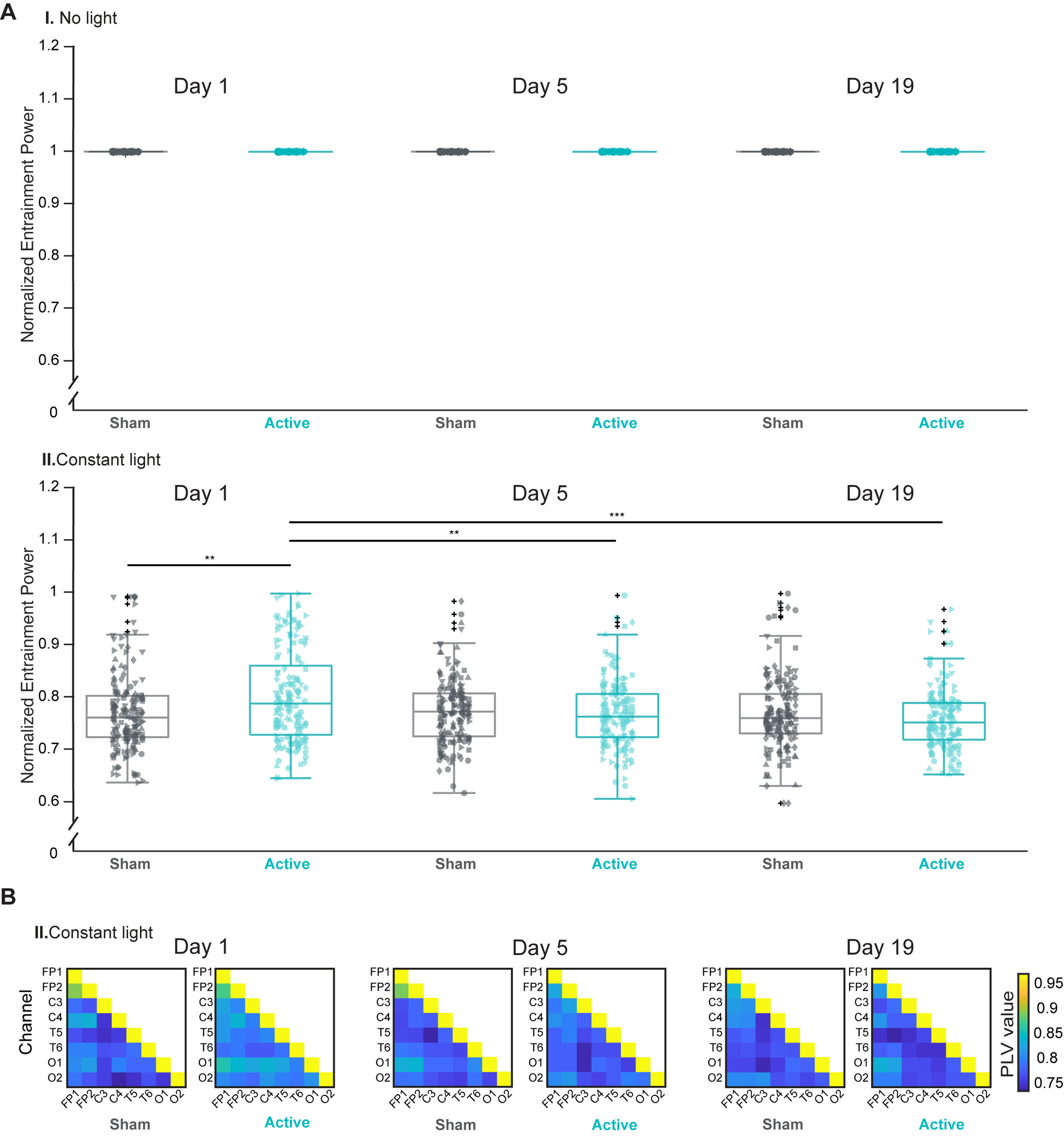

Supplement: S6 Fig — (A) Significant differences in normalized PLV (normalized to PLV matrix of No Light condition) were observed across all channel pairs between the active and sham groups under Constant light conditions on days 1. Inter-group comparisons were assessed using the Wilcoxon rank-sum test. Significant differences were observed in PLV measurements for the active group between day 1 and days 5 and 19. Intra-group comparisons across days were evaluated using the Kruskal-Wallis test, followed by post-hoc pairwise comparisons performed using Dunn’s test with Bonferroni correction. The statistically significant differences in PLV observed under constant light conditions are likely attributable to the large number of channel pair comparisons (n) rather than meaningful biological variation, since the average PLV values across groups and days remain comparable. Normality of the data was assessed using the Shapiro-Wilk test, and non-parametric methods were employed due to deviations from normality. (B) PLV matrices for the active and sham groups across experimental days under (I.) No light and (II.) Constant light conditions. Each element in the matrix represents the PLV for specific pairs of EEG channels, with diagonal elements showing a value of 1, indicating PLV between identical signals. Significant differences are indicated, with ** indicating p < 0.01 and *** indicating p < 0.001. Detailed p-values are provided in S3 Table. (TIF) [file pone.0332310.s009.tif]

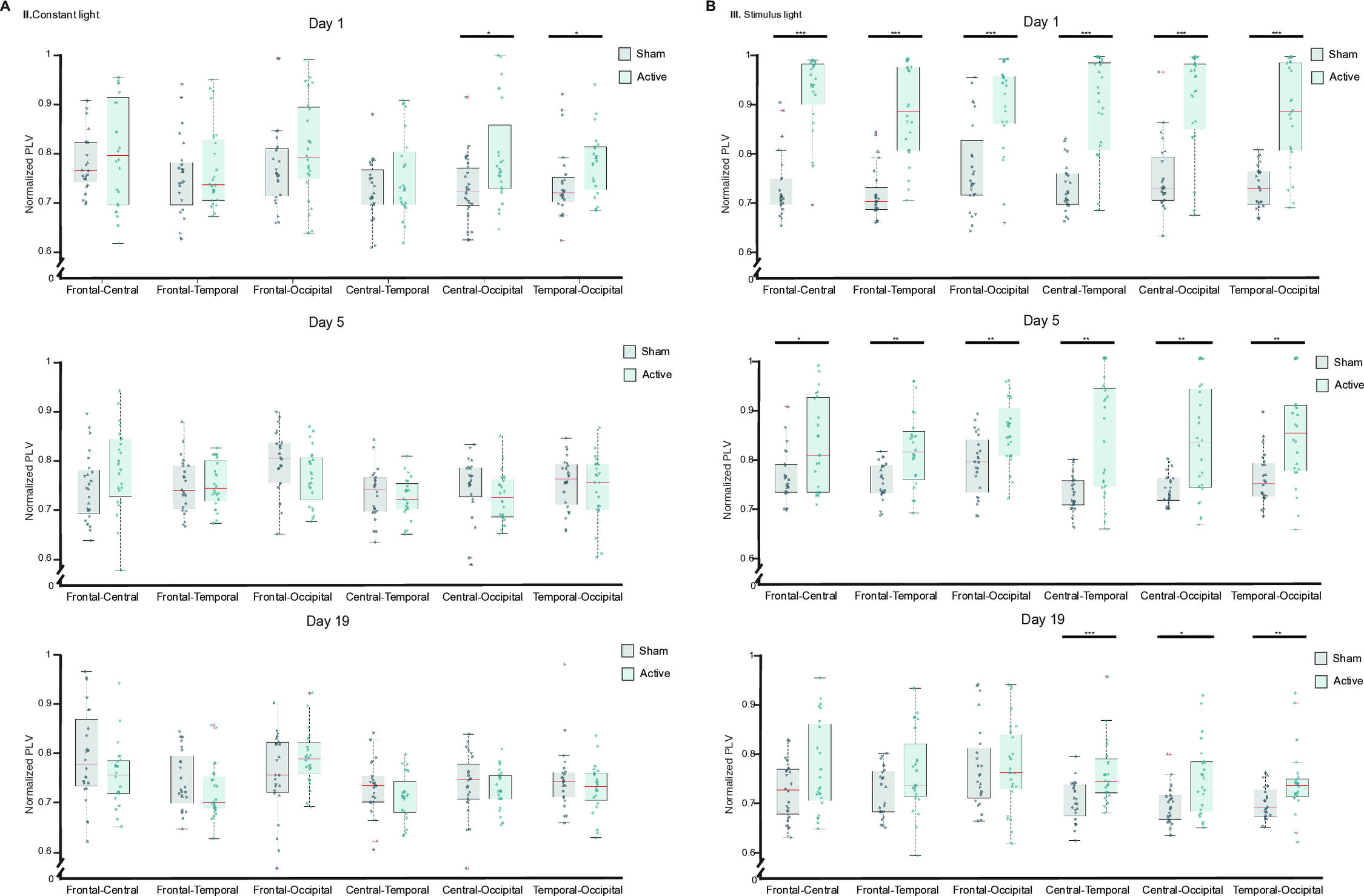

Supplement: S7 Fig — Box blots depicting normalized phase-locking value (PLV) at 60 Hz between the frontal, temporal, central, and occipital regions on days 1, 5, and 19. This highlights the spatial patterns of phase synchronization during repeated stimulation sessions. (A) shows PLV values during constant light conditions, and (B) shows PLV values during stimulus light conditions (60 Hz flicker for the active group and constant light for the sham group). Statistical significance was assessed using the Wilcoxon rank-sum test. Normality of the data was assessed using the Shapiro-Wilk test, and non-parametric methods were employed due to deviations from normality. Significant differences are indicated with *p < 0.05, **p < 0.01, and ***p < 0.001. Detailed p-values are provided in S3 Table. (TIF) [file pone.0332310.s010.tif]
