# Supplementary material for: Exploring neural entrainment and synchrony in response to repeated 60 Hz flickering white light in healthy volunteers
Source: PLoS One. 2025 Oct 7;20(10):e0332310. doi: 10.1371/journal.pone.0332310 (PMC12503310; doi:10.1371/journal.pone.0332310)
Supplement: Attachment — Submitted filename: Response to Reviewers_Alamalhoda et al.docx [file pone.0332310.s011.docx]

**Dr Gennady S. Cymbalyuk**

**Academic Editor**

**PLOS ONE**

Dear Dr Cymbalyuk,

We are very grateful for your work and the efforts of the Reviewers, and we are pleased that the manuscript was found to have merit. This rebuttal letter contains the responses to each point raised by the editorial team as well as the Reviewers.

A. **Editorial requirements**

Following your requests, we have made the following changes to the manuscript and accompaning files:

1. We have ensured that the manuscript meets PLOS ONE's style requirements, including those for file naming.

2. We have updated the financial disclosure to clarify the role of Funders, especially that of Syntropic Medical. This is our current discolosure:

*This study was funded by Syntropic Medical and supported by an Austria Wirtschaftsservice (AWS) grant (grant number P2414247 to Syntropic Medical). Syntropic Medical employees were involved in study design, data collection and analysis, decision to publish, and preparation of the manuscript. AWS had no role in study design, data collection and analysis, decision to publish, or preparation of the manuscript.*

3. We have removed the Austrian Research Promotion Agency (FFG) from our Acknowledgments section (this agency has not directly supported this project but has been key to other ones; we thought it would be kind to thank them here but we understand this creates confusion so we decided to remove them altogether). No update to the Funding section is needed.

4. Following your instructions and an email exchange with PLOS ONE publication assistant Mr Jacob Ulmschneider (June 6th) we have decided on a data sharing plan and clarified this in the current manuscript. Our plan is to make data available after acceptance, by simply populating an already existing JitHub link with the pseudonymized data we have. This is not a long process and will be done immediately after acceptance. This is now specified in the text as follows:

*´The EEG data and custom code used for preprocessing data and analysis will be made available upon acceptance at Github.com/AminAlam/HVS.´*

5.  We now include a list of Supplementary Files with titles and captions at the end of the manuscript, and updated any in-text citations to match accordingly.

**B. Point-by-point rebuttal to Reviewers' comments:**

**Reviewer #1**

**Major comments**:

1. The choice of 60 Hz for stimulation requires clearer justification. Given the growing body of evidence supporting 40 Hz entrainment in modulation neural activity, a comparison between 40 Hz and 60 Hz stimulation would provide a more informative perspective. The rationale for selecting 60 Hz should be explicitly discussed, particularly in relation to known effects.

We thank the Reviewer for this important comment. In the revised introduction, we clarified and expanded the rationale for selecting 60 Hz stimulation and included relevant citations demonstrating its emerging significance. Specifically, we now reference recent studies using 60 Hz transcranial alternating current stimulation (tACS) and auditory stimulation in humans, including those by:

Manippa et al. (2024), who reported reduced intrusion errors and enhanced cognitive control in healthy adults following 60 Hz auditory stimulation,

Nomura et al. (2019), who demonstrated that 60 Hz tACS over the dorsolateral prefrontal cortex (DLPFC) enhances declarative long-term memory (LTM).

These additions provide context regarding the functional relevance of 60 Hz gamma-band activity in executive and memory-related processes and address the concern that the original text lacked supporting human data.

Further, we emphasize that while 60 Hz has been tested in auditory and electrical protocols, its use in visual entrainment, especially over multiple weeks, remains largely unaddressed. Our study aims to explore this area. This point has been explicitly stated in the revised introduction (page 5 lines 97-105).

We agree that a lot is known on the effects of 40 Hz and a direct comparison with 60 Hz would be interesting to perform. Our goal for the present study was to generate a knowledge base with a full characterization of 60 Hz light stimulation over time – something that is missing in the literature. As a subsequent study, a direct comparison with 40 Hz would be highly relevant. To acknowledge this, in the Limitations section, we added a paragraph acknowledging the limitations of our study, such as the absence of 40 Hz for direct comparison of effects, as well as the broader absence of comparable human data on the effects of different frequency visual stimulation. By clearly outlining these gaps and open questions, we hope to facilitate future research on frequency-specific neuromodulation.

2. The final sample size (n = 6 per group after dropouts) is small, particularly for EEG-based study on younger adults. Furthermore, the observed subject variability in entrainment strength across the 3 EEG sessions makes drawing any strong conclusions about the habituation effect difficult.

We acknowledge the Reviewer's concerns about the small sample size and its implications for statistical power. Our study was designed (and approved by the Ethics Commission of Lower Austria) to be an initial, pilot investigation to evaluate the feasibility, safety and neurological effects of 60 Hz flickering light stimulation over an extended period, which was never done before in the literature. Such a small sample size has been previously used in some EEG studies, such as Sharpe et al., 2020 and Mangia et al., PLOS ONE 2014 (now quoted).

We also agree with the Reviewer on the high variability in entrainment strength, which limits our ability to make strong conclusions about habituation. In response, we have revised the manuscript to temper these claims, emphasising the exploratory nature of our findings and the necessity for larger-scale studies to validate and extend our results. These clarifications have been incorporated into the abstract (page 2, line 40-43 and 48-49) and discussion section (pages 31- 35, sections 4.2 to 4.5, and especially 4.6 limitations pages 36-37). We have also modified the title accordingly, which now reads ´*Exploring Neural Entrainment and Synchrony in Response to Repeated 60 Hz Flickering White Light in Healthy Volunteers*´.

3. It is unclear whether light intensity was adjusted on each day and how this was accounted for in the analyses. If intensity varied across days or between participants, this could have confounded the results. As stimulus intensity can influence the response, it is important to confirm that the total stimulus power was comparable between sessions and conditions. Without this control, it is difficult to determine whether reduced entrainment strength is due to habituation or changes in stimulus intensity.

We thank the Reviewer for this valuable feedback, indeed this is an important point to clarify. The stimulation protocol was designed to ensure safety and comfort, allowing for individualised light intensity settings (within a given range) based on verbal feedback from participants. To assess whether the intensity chosen could influence the entrainment and habituation observed, we have now performed an additional analysis to explore the changes in intensity levels selected by participants across the days. This analysis (presented in SI 1Fig D-E) revealed that participants chose an average light intensity of approximately 80 μW (SD: 7 μW). Notably, intra-individual variation over the 15 stimulation days was minimal, with a maximum SD of 9 μW for any single participant, representing less than 11% of the mean intensity. No decline in light intensity was observed over the days of stimulation, suggesting that this is not a factor involved in the observed habituation. Given this consistency across subjects and days, and the absence of a reduction in intensity over time, we believe it is unlikely that small day-to-day variations in stimulus power explain the observed reduction in entrainment strength over time. This important point has now been clarified in both the Methods and Discussion sections.

4. The Methods section lacks a description of EEG preprocessing and analysis steps, which are critical for interpreting the EEG findings. Additionally, the questionnaires used for assessing tolerability and other outcomes are not described. These methodological omissions should be addressed to ensure reproducibility and transparency.

We thank the Reviewer for this valuable observations regarding the Materials and Methods section, giving us an opportunity to improve its readability and transparency. We agree that the choice to place some of the key information in Supplementary might not have been ideal, so we have significantly restructured the M&M section accordingly. First, we have incorporated the supplementary materials and methods section into the main text – this includes thorough details on EEG preprocessing (Page 11 lines 248-260) and tolerability and blinding questionnaires (page 16, lines 353-367). In addition, to increase readability, in the revised manuscript, we have thoroughly restructured several paragraphs, including the Signal Processing and Analysis section, and added clearly labeled subheadings for each step of the EEG pipeline: “EEG Preprocessing,” “FFT Analysis,” “Topoplots,” “STFT,” and “PLV Calculation.”. Also, addressing Minor Comment 2 below, we have moved the technical details (manufacturer´s names and catalogue numbers of device components) to a separate table (SI Table 2), to improve the flow.

5. The manuscript would benefit from additional analysis of the temporal dynamics of the observed effects (comparing pre-, during, and post-stimulation). This would provide additional insight into whether such stimulation has transient or longer-lasting effects.

We thank the Reviewer for this suggestion regarding a further exploration of our data to assess the temporal dynamics of neural entrainment.

Each EEG session included three consecutive recording phases: a five-minute baseline period (no light), a five-minute period of constant light exposure, and a 20-minute stimulation phase (60-Hz flickering light or constant light). This design enabled us to compare neural activity pre-stimulation and during stimulation within each session.

Following the Reviewer’s comment, we have performed an additional temporal analysis to characterize the dynamics of entrainment during stimulation with more granularity. Specifically, we have divided the 20 min stimulation period into five non-overlapping, 4 minute intervals and analyzed the normalized 60-Hz power within each segment. The results showed that entrainment strength remained stable throughout the 20-minute stimulation phase, across all three days (Day 1, Day 5, and Day 19). This suggests sustained entrainment, with no evidence of decay or rapid adaptation during the stimulation period. This new analysis is included as SI Fig 3.

However, we acknowledge that our protocol did not include EEG recordings after the stimulation phase. Consequently, we cannot directly assess post-stimulation effects or the potential persistence of entrainment. We now explicitly state this limitation in the Results section (page 33, lines 755-758). The Results paragraph now reads:

*Our experimental design allowed us to study in detail the changes in entrainment power comparing pre-stimulation and during stimulation conditions. We must note that post-stimulation effects or the potential persistence of entrainment were not explored in this experiment and would be the subject of subsequent studies.*

6. Demographic comparisons appear to have been conducted on the full sample, but two participants in the Active group were excluded from EEG and biomarker analysis. It would be helpful to clarify whether the demographic balance remained after these dropouts, especially since the final analysis excluded their data.

We thank the Reviewer for highlighting this important aspect. Although initially conducted on the full randomized sample (N = 14), we agree that it is important to assess demographic balance within the final analysis cohort (N = 12), which comprises only participants with complete EEG and biomarker data. A re-analysis of the data was therefore performed. As a result we found that the 2 drop outs did not substantially affect the group balance, since there were no statistically significant differences between the groups in the final analysis sample in terms of age, sex, race or years of education. Updated group statistics and comparisons are included now in Table 1 for transparency and a clarification has been added in the accompanying text and caption (page 18) as follows:

*Two participants in the active group discontinued the study for personal reasons. Because of incomplete data, their EEG and biochemical measurements were excluded from the data analysis. However, both individuals completed the final questionnaire, and their responses on subjective tolerability, blinding and side effects were included in the relevant analyses. The groups remained balanced also after the two dropouts (Table 1).*

7. The authors mention that statistical test selection was based on the outcome of normality tests. However, repeated measures were analyzed with two-way ANOVA, which assumes normality. It should be clarified whether ANOVA was applied only to variables that passed normality checks, or if a non-parametric alternative was considered when assumptions were violated.

We thank the Reviewer for pointing this to us. We are sorry that our text in the Statistic section was not clear in this regard. Indeed all data have been tested for normality and parametric tests have been used only when the normality was ensured. We have now edited the section (page 17) to clarify this, as follows:

*´Statistical analysis for biomarker, demographic and questionnaire data were performed using GraphPad (versions 5 and 10.4) The Shapiro-Wilk test was used to assess the normality of the data. Age and years of education did not show a normal distribution and were analyzed with a Wilcoxon rank-sum test. Cortisol levels followed a normal distribution and were analyzed using a two-way ANOVA to evaluate the interaction between group (active versus sham) and time. Since CRP levels did not meet the normality assumption, they were analyzed using the Kruskal–Wallis test. Ordinal questionnaire responses were evaluated using the Wilcoxon rank-sum test. Proportions (race, sex and blinding efficacy) were compared using Fisher's exact test. A significance threshold of p < 0.05 was applied to all tests.´*

8. The Discussion references “quality-of-life” assessments, but these are not clearly defined or discussed elsewhere in the manuscript. It is important to distinguish between assessing tolerability/safety and evaluating quality of life, as these represent different outcomes.

We thank the Reviewer for highlighting this distinction. We acknowledge that the term 'quality of life' was used inaccurately in the Discussion, as we did not administer any standardised instruments that would enable us to assess quality of life. In our study, we collected subjective reports related to tolerability and perceived well-being, including minor side effects, willingness to continue stimulation and overall participant impressions.

To address this, we have revised the relevant sentence in the Discussion section to reflect the scope of the data collected. The term 'quality of life' has been replaced with a more appropriate description of tolerability and subjective experience (page 35).

**Reviewer 1 Minor comments:**

1. Several sentences should be revised. E.g., “Additional infos on light stimulation device and the mini-Faraday cage in Supplementary Methods”, “Additional infos and EEG data analysis in Supplementary Methods”.

Thanks for this comment. We have revised the text to reflect the fact that the additional methods are now in the main manuscript text, and edited those sentences accordingly.

2. Some sections include excessive technical detail (e.g. manufacturer part numbers, catalog numbers) that could be moved to the Supplementary Materials to improve readability and flow.

We concur with the Reviewer and have moved the technical details (manufacture´s names and catalogue numbers for device components) to a separate Supplementary table (SI Table 2), to improve the flow and readability.

**Reviewer #2**

**General comments**

1. The choice of 60 Hz is not sufficiently justified. One of the reasons the authors cite is that this frequency was not explored in humans before, but they fail to cite other studies using 60 Hz sensory stimulation who also compare effects of other frequencies (e.g., Manippa et al., 2024) and their effects on task performance. This set of studies suggested a positive effect of 60 Hz auditory stimulation on executive functions such as cognitive control and attention (not memory).

Thanks for this important comment. We acknowledge that the rationale for chosing 60 Hz might have not been sufficiently explained in the original version of the manuscript. In the revised introduction, we now clarify and expand the rationale for selecting 60 Hz. We emphasize that while 60 Hz has been tested in auditory and electrical protocols, its use in visual entrainment, especially over multiple weeks, remains largely unaddressed. Our study aims to explore this area. This point has been explicitly stated in the revised introduction (page 5 lines 97-105).

We also thank the Reviewer for pointing us to key references in this field. These are very useful to provide context to our findings and support an interesting role of 60 Hz modulation in humans. Accordingly, we now reference:

Manippa et al. (2024), who reported reduced intrusion errors and enhanced cognitive control in healthy adults following 60 Hz auditory stimulation,

Nomura et al. (2019) who demonstrated that 60 Hz tACS over the dorsolateral prefrontal cortex (DLPFC) enhances declarative long-term memory (LTM).

We still note that much less literature exists on 60 Hz entrainment (especially driven by flickering light) as compared to 40 Hz, which has been extensively studied. Given its functional relevance in executive and memory related processes, its role in PNN remodelling (based on our preclinical data) and the paucity of data currently existing, we think there is a strong rationale for exploring the effects on 60 Hz in humans further.

2. At least one lower frequency should have been tested as a control. In the cited animal study (Venturino et al., 2021), 60 Hz—compared to 40 Hz—had a specific effect on PNN/microglia, which could have been directly contrasted.

We thank the Reviewer for this valuable feedback. We agree that including a lower-frequency control condition, such as 40 Hz, which has well-documented effects on neural activity and microglial response, would have added significant value to our study and enabled direct, frequency-based comparisons.

However, the primary goal of this pilot study was to assess the feasibility and tolerability of 60 Hz flickering light, as well as its preliminary neural effects in humans. As such, we limited the scope to a single stimulation frequency. In the revised manuscript (page 37, line 835-838), we now explicitly acknowledge the absence of a 40 Hz condition as a limitation of our study, and emphasise the need for future studies to directly compare the effects of different gamma frequencies, including both 40 Hz and 60 Hz, to better contextualise our findings, as follows:

*Fourth, the absence of a 40 Hz control condition limits the interpretability of the frequency-specific effects observed. While our primary aim was to investigate the feasibility and tolerability of 60 Hz flickering light, future studies should include direct frequency comparisons to better contextualize these findings.*

3. The number of participants who completed the EEG study is too low, with only 6 in the active group. Since the protcol is not harmful, this low number might be reconsidered. Maybe, because of that, the data also seem under-analysed with regard to effects within individuals over time, and analysis of differences between electrodes. Given that there are reports that gamma stimulation does not even reach the hippocampus that point might be important to adress.

We thank the Reviewer for this important comment. We acknowledge that the small sample size (n = 6) is a limitation of our study. This limitation has been explicitly acknowledged in the revised manuscript (Limitations section, page 36, lines 812-820). The current study was designed as a pilot to assess the feasibility, tolerability, and basic neural effects of 60 Hz flickering light stimulation in humans. The focus was on establishing safety and detecting evidence of entrainment. To contextualize the use of small samples (n<10) in early-stage neurostimulation research, we now cite Sharpe et al., 2020 and Mangia et al PLOS ONE 2014.

Regarding the individual- and electrode-level analyses, we appreciate the suggestion and have performed additional analysis to address it. We have now included electrode-wise analyses and intra-subject comparisons across time points to address this issue more comprehensively. The results of these analyses are presented in the new SI Fig 4, 5, and 7 and are referenced in the Results section. Importantly, thanks to this additional analysis, we now report that we have not observed a specific topographic pattern of entrainment/synchronization nor in their decline.

Regarding the concern that gamma stimulation may not reach deeper brain structures, such as the hippocampus, we agree that this is a critical and currently debated point in the field (Suk et al. 2020 vs Soula et al., 2023). However, our current experiemntal design is not suited to investigate subcortical activity, as our EEG setup captures cortical surface activity, where entrainment effects can be robustly detected. For this reason we do not refer to hippocampal engagement in the paper.

4. The protocol does not appear fully consistent. What was the reason for not stimulating on weekends? One would expect daily stimulation to observe adaptation dynamics more precisely.

We thank the Reviewer for raising this point. The decision to exclude weekend stimulation was based on logistical considerations: the participants were healthy volunteers with weekday schedules, and requiring them to attend at the weekend could have increased the risk of non-compliance or dropout. We have now added this point to page 20 line 430-32.

Furthermore, it is common in multi-week neuromodulation protocols to limit sessions to weekdays, as demonstrated in previous studies with various modalities including tDCS and tACS (e.g. Woodham et al, Nature Medicine 2025, Gall et al., 2016 PLOS ONE 2016). Our protocol maintained a consistent schedule of five days per week across three weeks to support adherence.

However, we agree that uninterrupted daily stimulation, including weekends, may have provided a more continuous observation of adaptation dynamics.

5. EEG recordings should have been conducted on consecutive days during at least one week to investigate the timeline of entrainment decline. For example, was there any recovery over the weekend, or was the decline sharper during consecutive days?

We thank the Reviewer for this important point. We agree that consecutive EEG recordings might have been more informative but given the pilot nature of this study, we chose to examine entrainment over three time points only. In future studies a more frequent measurement with additional follow ups might be very interesting. With the data we have unfortunately we are not able to address the question of a potential recovery over the weekend, as we lack EEG recordings done on consecutive Fridays and Mondays (EEG was done on Monday-Friday on week 1 and then Friday on the week 3).

6. It remains unclear how long the adaptation effect lasted after the flicker stimulation ended. One could have included a follow-up.

We agree with the Reviewer that the timeline of the neural adaptation is of utmost interest. Our preliminary data provide initial indications on it but more detailed studies will be needed to explore this phenomenon in the future. The design of our study did not include any follow-up, as it was a pilot experiment mostly aimed at ensuring safety of the stimulation and to gather an initial idea on the entrainment effects in humans. We agree this is a limitation of the study and we mention it in our discussion as such.

7. There are references missing on the impact of sensory gamma stimulation on the glymphatic system.

We thank the Reviewer for pointing to this important aspect of 40 Hz effects. We have now added a reference to this topic in the introduction (Murdock et al 2024, Nature).

**Detailed comments**

1. Figure 1G: which electrode is shown? Please show at least two different ones.

We thank the Reviewer for pointing out this clarification as the original figure legend might have been misleading. Figure 1G does not represent data from a single electrode; rather, it shows the mean signal across all electrodes from one representative participant (active). This has now been clearly stated in the revised figure legend.

To better illustrate inter-electrode variability, we have included an additional panel showing the standard deviation of the signal across electrodes for the same participants and time point. This new panel has been added as Supplementary Figure 5.

2. Although the overall analysis suggests entrainment and adaptation across all electrodes, there seem to be considerable differences between them as suggested by the images. These differences should be quantified (for example, in Figure 2A, active group day 1).

Thanks for this thoughtful suggestion; channel-specific entrainment and adaptation patterns would be indeed a very interesting finding. To address this we have performed additional analysis of our data, grouping the channels according to topography in: occipital region, (O1 and O2), temporal region (Tp5 and Tp6), central region (C3 and C4) and frontal region (Fp1 and Fp2). All electrodes seem to show entrainment and we have not found any specific pattern of entrainmnet or adaptation, suggesting that both occur homogeneously	throughout the brain in the time-scale we can measure. However, as the Reviewer pointed out, we did find quite significant variability, in particular in the temporal area, which had the widest SD of all. We have performed a similar analysis also for the PLV data, without	finding any difference. These analyses are now featured in SI Fig 4 and 7.

3. A comment should be made on why the frontal or temporal electrodes seem to adapt more than the occipital ones, which are closer to the flickering visual input.

We thank the Reviewer for highlighting this observation. In response, we have added a statement to the discussion clarifying that the apparent greater adaptation in frontal or temporal electrodes is likely not biologically meaningful. Rather, this pattern appears to be primarily driven by variability between electrodes and subjects, as demonstrated by the detailed analysis of entrainment strength across brain regions over time and stimulation conditions (see SI Figure 4). We have noted this in the manuscript to help avoid overinterpretation of the spatial distribution of entrainment decline. The paragraph reads (page 23, line 526-529):

*This increase was observed across frontal, central, temporal, and occipital regions, indicating widespread neural entrainment. We did not observe any topographically specific pattern of decline, with signal decreasing homogeneously across all channels over the 19 days (SI Fig 4); however, the temporal channels showed the greatest variability across the 3 recording days.*

4. A discussion and citations should be included on which stimulation frequencies are able to induce entrainment in the visual system.

We thank the Reviewer for this suggestion. In the revised Discussion, we have added a short paragraph summarizing current knowledge on frequency-dependent visual entrainment (page 30, lines 664-671). We now cite key reviews and experimental studies that have characterized frequency-specific entrainment in the visual cortex (e.g., Herrmann 2001; Norcia et al. 2015, Gulbinaite et al. 2017) and discuss how our findings at 60 Hz fit within this broader framework.

5. How much decline in 60 Hz entrainment occurred during a single 20-minute session after 5 and 19 days?

Thanks for this interesting question. Following the Reviewers´ comments we have performed an additional temporal analysis to characterize the dynamics of entrainment during stimulation, with more granularity. Specifically, we have divided the 20 min stimulation period into five non-overlapping, 4 minute intervals and analyzed the normalized 60-Hz power within each segment. The results showed that entrainment strength remained stable throughout the 20-minute stimulation phase, across all three days (Day 1, Day 5, and Day 19). This suggests sustained entrainment, with no evidence of decay or rapid adaptation during the stimulation period. This new analysis is included as SI Figure 3.
