# Supplementary material for: Exploring neural entrainment and synchrony in response to repeated 60 Hz flickering white light in healthy volunteers
Source: PLoS One. 2025 Oct 7;20(10):e0332310. doi: 10.1371/journal.pone.0332310 (PMC12503310; doi:10.1371/journal.pone.0332310)
Supplement: Attachment — Submitted filename: Second Response to Reviewers_Alamalhoda et al.docx [file pone.0332310.s012.docx]

**Dr Gennady S. Cymbalyuk**

**Academic Editor**

**PLOS ONE**

Dear Dr Cymbalyuk,

We are delighted to see that the Reviewers´concerns have been sufficiently addressed and the revised version of the manuscript was found to have merit. Here below you´ll find the responses to the minor points raised.

- In the abstract (line 35), there appears to be a typo: "flicking light across multiple cortical regions" should likely read "flickering light".

We thank the Reviewer for highlighting this typo. This has been corrected.

- The naming of temporal channels is inconsistent throughout the manuscript and figures (e.g., sometimes Tp7-Tp8, Tp5-Tp6, or T5-T6). Please, unify this notation.

Thanks for pointing us to this inconsistency. We have now edited the text throughout the manuscript, using T5-T6 to refer to temporal channels.

- In the caption for SI Figure 6 (lines 1425-1427), the statement "The observed statistically significant differences are driven primarily by the large sample size (n), rather than any meaningful biological variation..." could be misleading. Please, clarify this point in the text to avoid confusion.

We concur with the Reviewer that this sentence might have been misleading. We have now edited as follows: *´The statistically significant differences in PLV observed under constant light conditions are likely attributable to the large number of channel pair comparisons (n) rather than meaningful biological variation, since the average PLV values across groups and days remain comparable.´*

Please note that we have run an additional spelling and reference check and corrected a few minor mistakes in this revised version. We hope the revised paper meets PLOS ONE publication criteria and we remain at your disposal for any clarification needed.

Sincerely,

[Maria Teresa Ferretti](mailto:mariateresa@syntropicmedical.com) for the co-authors

Vienna, August 25th 2025
